# LDL-cholesterol and body mass index among Japanese schoolchildren: a population-based cross-sectional study

**DOI:** 10.1186/1476-511X-12-77

**Published:** 2013-05-24

**Authors:** Takako Shirasawa, Hirotaka Ochiai, Tadahiro Ohtsu, Rimei Nishimura, Aya Morimoto, Hiromi Hoshino, Naoko Tajima, Akatsuki Kokaze

**Affiliations:** 1 Department of Public Health, Showa University School of Medicine, 1-5-8 Hatanodai, Shinagawa-ku, Tokyo 142-8555, Japan; 2 Division of Diabetes, Metabolism and Endocrinology, Department of Internal Medicine, Jikei University School of Medicine, Tokyo, Japan; 3 Jikei University School of Medicine, Tokyo, Japan

**Keywords:** Serum low-density lipoprotein, Body mass index, Schoolchildren

## Abstract

**Background:**

Serum low-density lipoprotein cholesterol (LDL-C) is one of the most important risk factors for coronary heart disease. The aim of the present study was to investigate the relationship between LDL-C and body mass index (BMI) in population-based Japanese schoolchildren.

**Methods:**

The subjects comprised all fourth graders and seventh graders in Ina Town, Saitama Prefecture, Japan, during 2002-2009. Information about each subject’s age, sex, and family history of hypercholesterolemia was collected using a self-administered questionnaire. The body height, weight, and LDL-C were measured for each child. LDL-C was measured using the direct method. According to the LDL-C criteria of the Japan Atherosclerosis Society, LDL-C level was categorized into three subgroups: acceptable, < 110 mg/dL; borderline, 110-139 mg/dL; and high, ≥ 140 mg/dL. Children with either borderline or high LDL-C level were considered to have high-normal LDL-C (HLDL-C).

**Results:**

Data from a total of 5869 subjects were analyzed. A higher BMI category was associated with a higher prevalence of HLDL-C regardless of sex or grade level (*P* < 0.05). When compared with the <50th percentile BMI category, the odds ratio (OR) for HLDL-C was statistically significant in the 75th to 84th percentile category of fourth-grade boys (OR: 1.95, 95% confidence interval (95% CI): 1.28-2.97), the 85th to 94th percentile of fourth-grade girls (2.52, 1.74-3.64), and the 85th to 94th percentile of seventh-grade boys (2.04, 1.31-3.20) and girls (1.90, 1.24-2.91).

**Conclusion:**

A statistically significant association between LDL-C levels and BMI was observed in Japanese school children.

## Background

Some studies have reported that serum cholesterol levels have increased among Japanese children [1,2]. Previous studies have reported that childhood serum lipid levels and body mass index (BMI) are strongly correlated with those levels in middle age [3,4]. Moreover, a higher BMI during childhood is associated with increased risk of coronary heart disease (CHD) [5]. Therefore, it is very important to prevent childhood obesity, which may help prevent atherosclerosis and CHD in the future.

Serum low-density lipoprotein cholesterol (LDL-C) has been reported to be one of the most important risk factors of CHD [6,7]. Some studies have reported that an association of BMI with total cholesterol (TC) or high-density lipoprotein cholesterol (HDL-C) among children [1,2,8]. However, LDL-C levels have not been measured often among Japanese children at the population level [9,10].

The determination of serum LDL-C level is generally done using the Friedewald calculation, for which fasting serum triglyceride (TG) level is needed [11]. However, it is often difficult to obtain a fasting lipid profile in large population surveys [12,13]. Thus, some assays for the direct determination of serum LDL-C level that can be used with non-fasting serum samples have been developed [14]. Since this method is applicable to non-fasting serum samples [15,16], it is useful for the evaluation of dyslipidemia in healthy schoolchildren.

Accordingly, the aim of the present study was to investigate the relationship between LDL-C measured using the direct method and BMI in population-based Japanese schoolchildren.

## Methods

### Subjects and setting

As a part of its community health services, the town of Ina in Saitama Prefecture, Japan, has provided annual childhood health check-ups to help prevent lifestyle-related diseases since 1994; studies of this program have been reported [17-22]. The present study was conducted as a part of the program.

The subjects of this study comprised all fourth graders (age 9-10 years) and all seventh graders (age 12-13 years) who lived in Ina between 2002 and 2009. Written informed consent was obtained from each child’s parent or guardian. The study protocol was approved by the Medical Ethics Committee of Showa University School of Medicine.

### Variables and their measurement

The following information was collected using a self-administered questionnaire completed by each child: sex, age, and exercise other than physical education (daily, sometimes, or none). Additionally, each subject’s parent or guardian was asked to complete a self-administered questionnaire about parental history of hypercholesterolemia (Yes or No).

Measurements of height and weight were performed annually during 2002-2009. The same examination protocol was used annually to ensure uniformity of quality and precision of assessment. For the measurements, all participants were asked to remove their shoes and socks, after which their height and weight were measured in increments of 0.1 cm and 0.1 kg, respectively, while they were wearing light clothing. BMI was calculated as body weight (kg) divided by the square of the height (m^2^).

Venous blood samples were drawn from the study subjects to measure serum levels of LDL-C. LDL-C was measured by routine automated laboratory methods. The levels of LDL-C were determined using the Cholesterol-LDL Kit (Daiichi Pure Chemicals, Japan) [17]. According to the LDL-C criteria of the Japan Atherosclerosis Society [9], the LDL-C level was categorized into three subgroups: acceptable, < 110 mg/dL; borderline, 110-139 mg/dL; or high, ≥ 140 mg/dL. Children with either borderline or high LDL-C level were considered to have high-normal LDL-C (HLDL-C). BMI was classified into five categories (< 50th, 50th to 74th, 75th to 84th, 85th to 94th, and ≥ 95th percentile) according to a previous study [18].

### Data analysis methods

The Mann-Whitney *U*-test or chi-squared test was used to compare characteristics between boys and girls. The normality of distribution was tested for each variable. In the stratified analysis by sex and grade, the relationship between LDL-C and BMI was investigated using the chi-squared test and a logistic regression model. A *P* value of less than 0.05 was considered statistically significant and 0.05 ≤ *P* < 0.1 was regarded as marginally significant. All data were analyzed using SPSS 16.0 J (IBM, Chicago, IL, USA).

## Results

Among 5950 subjects, 81 were excluded from the analysis because of refusal to participate or school absence. Thus, data from a total of 5869 subjects were analyzed. The participation rate was 98.6%.

Table 1 shows characteristics of the subjects. Of the 5869 children, 3168 were fourth graders (1645 boys and 1523 girls) and 2701 were seventh graders (1380 boys and 1321 girls), with median age of 9.0 and 12.0 years, respectively. Median BMIs were 16.6 and 16.3 kg/m^2^ for fourth-grade boys and girls, respectively, and 18.1 and 18.3 kg/m^2^ for seventh-grade boys and girls. The BMIs were significantly higher in boys than in girls regardless of grade (fourth grade, *P* < 0.001; seventh grade, *P* = 0.036). Median LDL-C levels for fourth-grade boys and girls were 90.0 and 95.0 mg/dL, respectively, whereas those for seventh-grade boys and girls were 86.0 and 93.0 mg/dL. In both grades, LDL-C was significantly higher in girls than in boys (*P* < 0.001). The proportion of HLDL-C in fourth-grade boys and girls was 18.8% and 24.9%, respectively, and for seventh-grade boys and girls 15.6% and 20.6%. The proportion was significantly higher in girls than in boys regardless of grade (fourth grade: *P* = 0.002, seventh grade: *P* < 0.001). The proportion of children with a family history of hypercholesterolemia (father and/or mother had a history of hypercholesterolemia) was 6.0% and 5.2% for fourth-grade boys and girls, respectively, whereas for seventh-grade boys and girls it was 5.8% and 5.7%. There were no statistically significant differences between boys and girls for family history of hypercholesterolemia in either grade (fourth grade: *P* = 0.193, seventh grade: *P* = 0.480). There was a statistically significant difference between boys and girls in amount of exercise in both grades (*P* < 0.001).

**Table 1:** Characteristics of subjects

	**Fourth graders (age 9-10 years)**					**Seventh graders (age 12-13 years)**				
	**Boys**		**Girls**		P - value*	**Boys**		**Girls**		P - value*
	**(n = 1,645)**		**(n = 1,523)**			**(n = 1,380)**		**(n = 1,321)**		
Age (years)	9.0	(9.4)	9.0	(9.4)	0.751	12.0	(12.3)	12.0	(12.3)	0.277
Height (cm)	134.9		134.7		0.787	155.1		153.0		< 0.001
Weight (kg)	30.2		29.5		< 0.001	43.8		43.3		0.065
BMI (kg/m^2^)	16.6		16.3		< 0.001	18.1		18.3		0.036
LDL-C (mg/dL)	90.0	(92.9)	95.0	(97.3)	< 0.001	86.0	(88.9)	93.0	(95.0)	< 0.001
< 110 (%)	81.2		75.1		0.002	84.5		79.3		< 0.001
110 - 139 (%)	15.2		21.6			12.8		17.3		
140 + (%)	3.6		3.3			2.8		3.3		
Family history of hypercholesterolemia (%)										
No	94.0		94.8		0.193	94.2		94.3		0.480
Yes	6.0		5.2			5.8		5.7		
Exercise (%)										
Daily	63.1		42.0		< 0.001	84.1		59.3		< 0.001
Sometimes	23.4		31.7			7.4		13.1		
None	13.5		26.2			8.6		27.7		

Data are expressed as a median (mean) or percentage (%).

*Mann-Whitney *U*-test or the chi-square test.

BMI, body mass index; LDL-C, low-density lipoprotein cholesterol.

The associations of LDL-C with BMI, family history of hypercholesterolemia, and exercise are shown in Table 2 (fourth graders) and Table 3 (seventh graders). A higher BMI category was associated with a higher proportion of HLDL-C regardless of sex or grade level (*P* < 0.05). Among fourth graders, the proportion of HLDL-C was higher in those with a family history of hypercholesterolemia than in those without a family history of hypercholesterolemia in both sexes (boys: *P* = 0.001, girls: *P* = 0.037). Among seventh graders, there was association between HLDL-C and a family history of hypercholesterolemia in girls (*P* = 0.043), whereas there was no association between these factors in boys. The proportion of HLDL-C was higher in those who did not exercise than in those who did exercise daily, although the difference was not statistically significant for sex or grade level.

**Table 2:** Association of LDL-C with BMI, family history of hypercholesterolemia, and exercise among fourth graders (age 9-10 years)

	**Boys (n = 1,645)**					**Girls (n = 1,523)**				
	**< 110**		**110 ≤**		P - value*	**< 110**		**110 ≤**		P - value*
BMI percentile										
< 50th	719	(87.5)	103	(12.5)	< 0.001	606	(79.4)	157	(20.6)	< 0.001
50 - 74th	345	(83.7)	67	(16.3)		292	(76.8)	88	(23.2)	
75 - 84th	129	(78.2)	36	(21.8)		113	(74.3)	39	(25.7)	
85 - 94th	105	(64.0)	59	(36.0)		92	(60.5)	60	(39.5)	
≥ 95th	38	(46.3)	44	(53.7)		41	(53.9)	35	(46.1)	
Family history of hypercholesterolemia										
No	1269	(82.0)	278	(18.0)	0.001	1092	(75.6)	352	(24.4)	0.037
Yes	67	(68.4)	31	(31.6)		52	(65.8)	27	(34.2)	
Exercise										
Daily	831	(82.6)	175	(17.4)	0.126	468	(75.7)	150	(24.3)	0.595
Sometimes	291	(78.0)	82	(22.0)		353	(75.6)	114	(24.4)	
None	171	(79.5)	44	(20.5)		282	(73.1)	104	(26.9)	

Data are expressed as number (%).

*The chi-square test.

**Table 3:** Association of LDL-C with BMI, family history of hypercholesterolemia, and exercise among seventh graders (age 12-13 years)

	**Boys (n = 1,380)**					**Girls (n = 1,321)**				
	**< 110**		**110 ≤**		P - value*	**< 110**		**110 ≤**		P - value*
BMI percentile										
< 50th	598	(86.7)	92	(13.3)	< 0.001	545	(82.5)	116	(17.5)	0.007
50 - 74th	301	(87.0)	45	(13.0)		263	(79.7)	67	(20.3)	
75 - 84th	120	(87.6)	17	(12.4)		100	(75.8)	32	(24.2)	
85 - 94th	105	(76.1)	33	(23.9)		94	(71.2)	38	(28.8)	
≥ 95th	42	(60.9)	27	(39.1)		46	(69.7)	20	(30.3)	
Family history of hypercholesterolemia										
No	1101	(84.7)	199	(15.3)	0.247	995	(79.9)	251	(20.1)	0.043
Yes	65	(81.2)	15	(18.8)		53	(70.7)	22	(29.3)	
Exercise										
Daily	945	(85.2)	164	(14.8)	0.054	616	(81.7)	138	(18.3)	0.100
Sometimes	76	(78.4)	21	(21.6)		129	(77.7)	37	(22.3)	
None	89	(78.8)	24	(21.2)		269	(76.4)	83	(23.6)	

Data are expressed as number (%).

*The chi-square test.

Logistic regression analysis was conducted to calculate odds ratios (ORs) for HLDL-C and their 95% confidence intervals (95% CIs) (Table 4). When compared to children in the < 50th percentile BMI category, significantly increased ORs were observed in the category of 75th to 84th percentile fourth-grade boys (OR: 1.95, 95% CI: 1.28-2.97), 85th to 94th percentile fourth-grade boys (3.92, 2.68-5.74) and girls (2.52, 1.74-3.64), and 85th to 94th percentile seventh-grade boys (2.04, 1.31-3.20) and girls (1.90, 1.24-2.91). In the ≥ 95th percentile category, the OR significantly increased regardless of grad level or sex. Moreover, regarding the association between HLDL-C and BMI, a significant dose-response relationship was observed regardless of grade level or sex (*P* for trend < 0.001). Even when we adjusted for family history of hypercholesterolemia and exercise in the analysis, the results remained the same. The interaction of BMI and family history of hypercholesterolemia on the presence of HLDL-C was not statistically significant. Although there was an interaction between BMI and exercise for the presence of HLDL-C among seventh-grade boys (*P* = 0.008), a significantly increased OR of BMI for HLDL-C was observed in the ≥ 95th percentile category for each exercise group (daily, sometimes, or none).

**Table 4:** Odds ratios and 95% confidence intervals for high-normal LDL-C by sex

		**Fourth graders (age 9-10 years)**					**Seventh graders (age 12-13 years)**				
			**Crude**		Adjusted*			**Crude**		Adjusted*	
		**n**	**OR**	**95% CI**	**OR**	**95% CI**	**n**	**OR**	**95% CI**	**OR**	**95% CI**
Boys											
BMI percentile											
	< 50th	822	1		1		690	1		1	
	50 - 74th	412	1.36	0.97-1.89	1.35	0.95-1.90	346	0.97	0.66-1.43	0.96	0.65-1.44
	75 - 84th	165	1.95	1.28-2.97	1.95	1.25-3.04	137	0.92	0.53-1.60	0.80	0.45-1.44
	85 - 94th	164	3.92	2.68-5.74	3.91	2.61-5.84	138	2.04	1.31-3.20	1.94	1.22-3.10
	≥ 95th	82	8.08	5.00-13.07	8.16	4.94-13.47	69	4.18	2.46-7.11	4.16	2.39-7.24
			(*P* for trend < 0.001)		(*P* for trend < 0.001)			(*P* for trend < 0.001)		(*P* for trend < 0.001)	
Girls											
BMI percentile											
	< 50th	763	1		1		661	1		1	
	50 - 74th	380	1.16	0.87-1.56	1.17	0.86-1.59	330	1.20	0.86-1.67	1.24	0.87-1.76
	75 - 84th	152	1.33	0.89-2.00	1.33	0.87-2.03	132	1.50	0.96-2.35	1.70	1.07-2.69
	85 - 94th	152	2.52	1.74-3.64	2.57	1.75-3.78	132	1.90	1.24-2.91	1.95	1.24-3.05
	≥ 95th	76	3.30	2.03-5.35	3.28	1.98-5.44	66	2.04	1.17-3.58	2.01	1.12-3.63
			(*P* for trend < 0.001)		(*P* for trend < 0.001)			(*P* for trend < 0.001)		(*P* for trend < 0.001)	

*Adjusted for family history of hypercholesterolemia and exercise.

BMI, body mass index; OR, odds ratio; 95% CI, 95% confidence interval.

## Discussion

In the present study, the relationship between LDL-C and BMI was investigated in a large sample of population-based Japanese schoolchildren. It was found that LDL-C measured using the direct method for fourth-grade boys and girls was 90.0 and 95.0 mg/dL, respectively, whereas for seventh-grade boys and girls the values were 86.0 and 93.0 mg/dL. These results were similar to those in previous studies using the direct measurement of LDL-C [15,16]. Moreover, in our study, LDL-C level was significantly higher in girls than in boys for both grades (*P* < 0.001). These results were consistent with previous nationwide reports among Japanese children [9,15]. The sex difference in the results may be explained in part by the influence of hormones on LDL-C among children [23,24]. Additionally, in our data, BMI was significantly higher in boys than in girls regardless of grade (*P* < 0.05). The previous study reported that sex differences in body composition are present very early in life, and are primarily attributable to the action of sex steroid hormones [25]. Accordingly, we analyzed the data separately for sex when the association between LDL-C and BMI was investigated.

A statistically significant association between LDL-C levels and a family history of hypercholesterolemia was observed among fourth graders. A previous study showed that, compared to adults, children with dyslipidemia had a stronger family or genetic background [26]. Moreover, in our study, there was a marginally significant difference between HLDL-C and exercise; physical exercise has been reported to be associated with LDL-C in children [27]. Therefore, we adjusted for these factors (a family history of hypercholesterolemia and exercise) to evaluate the relationship between LDL-C and BMI in schoolchildren.

In this study, a higher BMI category was associated with higher LDL-C levels regardless of grade or sex, which was consistent with previous studies [28-30]. Furthermore, the adjusted OR for high LDL-C among fourth graders and seventh graders reached a statistically significant level in the BMI category of greater than 85th percentile, which is classified as “overweight” if using the weight categories from the Centers for Disease Control and Prevention (CDC) [31]. Previous studies have reported that children who were obese had worse lipid profiles than those who were non-obese [27-29]. Murata showed that the incidence of childhood obesity and hyperlipidemia in Japan have increased year after year, and the reason was due to increased intake of fat and an increasingly sedentary lifestyle, including decrease in physical exercise and activity [1]. Furthermore, serum lipid levels and BMI in childhood correlate strongly with those values in middle age [3]. Our data showed that a BMI in the greater than 85th percentile category (ie, overweight) was associated with a higher LDL-C. Therefore, to reduce the presence of CHD risk factors, it is important to prevent children from becoming overweight. Intervention and education programs aimed at reducing overweight among schoolchildren might help improve LDL-C levels. Early lifestyle interventions could also help manage HLDL-C, contributing to primary prevention of CHD in the future.

The strength of this study is that the measurement of height, weight, and LDL-C level was conducted for about 6000 population-based schoolchildren. However, potential limitations should be discussed. First of all, the present study was a cross-sectional design. Therefore, a causal relationship between LDL-C and BMI cannot be determined. In the future, longitudinal research will be necessary to address this question. Second, the subjects in this study were children from only one town in Japan, which limits generalizability to other Japanese children or other races.

## Conclusions

In conclusion, the present study showed a statistically significant association between LDL-C levels and BMI in boys and girls in the fourth and seventh grades. Therefore, our study results suggest that it is important to prevent children from becoming overweight in order to help prevent the onset of hypercholesterolemia in the future.

## Competing interests

The authors declare that they have no competing interests.

## Authors’ contributions

TS, HO, and TO planned this study. RN and AM contributed to improving this study in a meaningful way. TS drafted this manuscript. HO, RN, and AM performed the data collection. HO was in charge of the supervision of the data collection. HH supported the data collection. HO and TO contributed to the statistical analysis. NT and AK made substantial contributions to the conception of this study and the revision of the manuscript. All authors read and approved the final manuscript.
